# Fuel for the work required: a practical approach to amalgamating train‐low paradigms for endurance athletes

**DOI:** 10.14814/phy2.12803

**Published:** 2016-05-24

**Authors:** Samuel G. Impey, Kelly M. Hammond, Sam O. Shepherd, Adam P. Sharples, Claire Stewart, Marie Limb, Kenneth Smith, Andrew Philp, Stewart Jeromson, D. Lee Hamilton, Graeme L. Close, James P. Morton

**Affiliations:** ^1^ Research Institute for Sport and Exercise SciencesLiverpool John Moores UniversityLiverpoolUK; ^2^ MRC‐ARUK Centre for Musculoskeletal AgingResearch Division of Medical Sciences and Graduate Entry MedicineSchool of Medicine Faculty of Medicine and Health SciencesUniversity of NottinghamRoyal Derby Hospital CentreDerbyUK; ^3^ MRC‐ARUK Centre for Musculoskeletal Aging ResearchSchool of Sport, Exercise and Rehabilitation SciencesUniversity of BirminghamBirminghamUK; ^4^ Health and Exercise Sciences Research GroupUniversity of StirlingStirlingUK

**Keywords:** Mitochondrial biogenesis, muscle glycogen, train‐low

## Abstract

Using an amalgamation of previously studied “train‐low” paradigms, we tested the effects of reduced carbohydrate (CHO) but high leucine availability on cell‐signaling responses associated with exercise‐induced regulation of mitochondrial biogenesis and muscle protein synthesis (MPS). In a repeated‐measures crossover design, 11 males completed an exhaustive cycling protocol with high CHO availability before, during, and after exercise (HIGH) or alternatively, low CHO but high protein (leucine enriched) availability (LOW + LEU). Muscle glycogen was different (*P* < 0.05) pre‐exercise (HIGH: 583 ± 158, LOW + LEU: 271 ± 85 mmol kg^−1^ dw) but decreased (*P* < 0.05) to comparable levels at exhaustion (≈100 mmol kg^−1^ dw). Despite differences (*P* < 0.05) in exercise capacity (HIGH: 158 ± 29, LOW + LEU: 100 ± 17 min), exercise induced (*P* < 0.05) comparable AMPK *α*2 (3–4‐fold) activity, PGC‐1*α* (13‐fold), p53 (2‐fold), Tfam (1.5‐fold), SIRT1 (1.5‐fold), Atrogin 1 (2‐fold), and MuRF1 (5‐fold) gene expression at 3 h post‐exercise. Exhaustive exercise suppressed p70S6K activity to comparable levels immediately post‐exercise (≈20 fmol min^−1^ mg^−1^). Despite elevated leucine availability post‐exercise, p70S6K activity remained suppressed (*P* < 0.05) 3 h post‐exercise in LOW + LEU (28 ± 14 fmol min^−1^ mg^−1^), whereas muscle glycogen resynthesis (40 mmol kg^−1^ dw h^−1^) was associated with elevated (*P* < 0.05) p70S6K activity in HIGH (53 ± 30 fmol min^−1^ mg^−1^). We conclude: (1) CHO restriction before and during exercise induces “work‐efficient” mitochondrial‐related cell signaling but; (2) post‐exercise CHO and energy restriction maintains p70S6K activity at basal levels despite feeding leucine‐enriched protein. Our data support the practical concept of “fuelling for the work required” as a potential strategy for which to amalgamate train‐low paradigms into periodized training programs.

## Introduction

Traditional nutritional strategies for endurance athletes have typically advised high carbohydrate (CHO) availability before, during, and after training sessions in order to support high daily training volume and intensities (Burke et al. 2011). However, in the last decade, accumulating data demonstrate that strategic periods of reduced CHO availability (the so‐called “train‐low” paradigm) actually augments selected skeletal muscle markers of training adaptation (Hawley and Morton 2014; Bartlett et al. 2015). For example, reducing endogenous and/or exogenous CHO availability during short‐term (e.g., 3–10 week) endurance training increases mitochondrial enzyme activity and protein content (Yeo et al. 2008; Morton et al. 2009; Van Proeyen et al. 2011), increases both whole body (Yeo et al. 2008) and intramuscular lipid oxidation (Hulston et al. 2010), and in some instances, improves exercise capacity and performance (Hansen et al. 2005; Cochran et al. 2015; Marquet et al. 2016). The augmented training responses observed when “training low” are thought to be mediated via the complex regulation of cell signaling pathways with potent roles in modulating an oxidative phenotype. Indeed, when exercise protocols are matched for work done, CHO restriction augments both AMPK (Wojtaszewski et al. 2003) and p38MAPK activation (Cochran et al. 2010) that ultimately converge on downstream transcription factors and coactivators such as PGC‐1*α* (Psilander et al. 2013), p53 (Bartlett et al. 2013), and PPAR*δ* (Philp et al. 2013). In the context of nutrient‐gene interactions, it is therefore apparent that the acute molecular regulation of cell signaling processes provides a theoretical basis for understanding the molecular mechanisms underpinning chronic training adaptations.

The research designs that have been used to study both acute and chronic train‐low adaptations thus far, have largely adopted twice per day training protocols (Hansen et al. 2005; Yeo et al. 2008; Hulston et al. 2010), fasted training (Van Proeyen et al. 2011), and CHO restriction during (Morton et al. 2009) and/or post‐exercise (Pilegaard et al. 2005). More recently, a “sleep‐low, train‐low” model has also been developed in which athletes perform an evening training session but sleep with reduced post‐exercise CHO intake, followed by completion of a fasted training session on the subsequent morning. Using this model, we (Bartlett et al. 2013) and others (Lane et al. 2015) observed enhanced activation of acute cell signaling pathways and expression of genes with putative roles in regulating training adaptation. Furthermore, when performed chronically as part of a periodized nutrition strategy, this model of CHO restriction also enhanced submaximal cycling efficiency, high‐intensity cycling capacity, and improved 10 km run time in already well‐trained triathletes (Marquet et al. 2016).

Despite the emergence of the aforementioned train‐low paradigms, the optimal approach for which to practically apply with athletic populations is not currently known. Such limitations are most well recognized for the potential reductions in absolute training intensity associated with reduced CHO availability (Widrick et al. 1993; Yeo et al. 2008; Hulston et al. 2010), perturbations to immune function and associated increases in muscle protein degradation (Lemon and Mullin 1980; Howarth et al. 2010), all of which could be detrimental to long‐term training and athletic performance. Furthermore, in the real‐world training environments of elite endurance athletes, it is likely that athletes practice an amalgamation of the aforementioned train‐low paradigms (either through default of their current training structure or via coach and sport scientist‐led practices), as opposed to undertaking one potential strategy in isolation. The complexity of practical train‐low models is also exacerbated by the observations that many endurance athletes (especially cyclists) also practice day‐to‐day or longer term periods of energy periodization (as opposed to CHO per se) in an attempt to reduce both body mass and fat mass in preparation for key competitive events (Stellingwerff 2012; Vogt et al. 2005; J.P. Morton, unpublished observations). Indeed, the performance improvements observed by Marquet et al. (2016) were also associated with a 1 kg reduction in fat mass induced by the periodized sleep‐low model. When taken together, such data highlight the requirement to study train‐low paradigms that may be more reflective of real‐world athletic practice (i.e., both CHO and energy restriction) and that are representative of an amalgamation of the train‐low protocols previously studied in the research setting.

With this in mind, we therefore examined the effects of high CHO versus low CHO availability on the modulation of those skeletal muscle cell‐signaling pathways with putative roles in the regulation of both mitochondrial biogenesis and muscle protein synthesis (MPS). We employed a repeated‐measures crossover design whereby healthy active males performed an exhaustive cycling‐based protocol in conditions of high CHO availability (i.e., “best” nutritional practice of CHO loading and CHO feeding during and after exercise) versus a nutritional protocol representative of both low CHO and energy availability (as achieved via 36 h of reduced CHO intake and omission of CHO intake before, during, and after exercise). In an attempt to compensate for the negative effects of energy deficit on muscle protein degradation and synthesis (Pasiakos et al. 2010, 2011, 2013; Breen et al. 2011; Areta et al. 2014), our low CHO protocol was also completed with leucine‐rich protein availability before, during, and after exercise. We specifically hypothesized that reduced CHO availability would impair exercise capacity but nonetheless, would induce comparable or superior mitochondrial‐related signaling thereby inducing a work‐efficient training and nutritional train‐low paradigm. We further hypothesized that high leucine availability would up‐regulate markers of protein synthesis and reduce markers of muscle protein breakdown.

## Materials and Methods

### Participants

Eleven recreationally active and amateur competitive male cyclists (age: 24.0 ± 3.3; height: 178 ± 10 cm; body mass: 79.6 ± 4.0 kg) who trained between 3 and 7 h per week took part in this study. Mean VO_2peak_ and peak power output (PPO) for the cohort were 53.6 ± 7.0 mL kg^−1^ min^−1^ and 285 ± 20 W, respectively. None of the participants had a history of neurological disease or skeletal muscle abnormality and none were under pharmacological intervention during the course of the study. All subjects provided informed written consent and all procedures conformed to the standards set by the Declaration of Helsinki (2008). The study was approved by the local Research Ethics Committee of Liverpool John Moores University.

### Design

**Figure 1:**
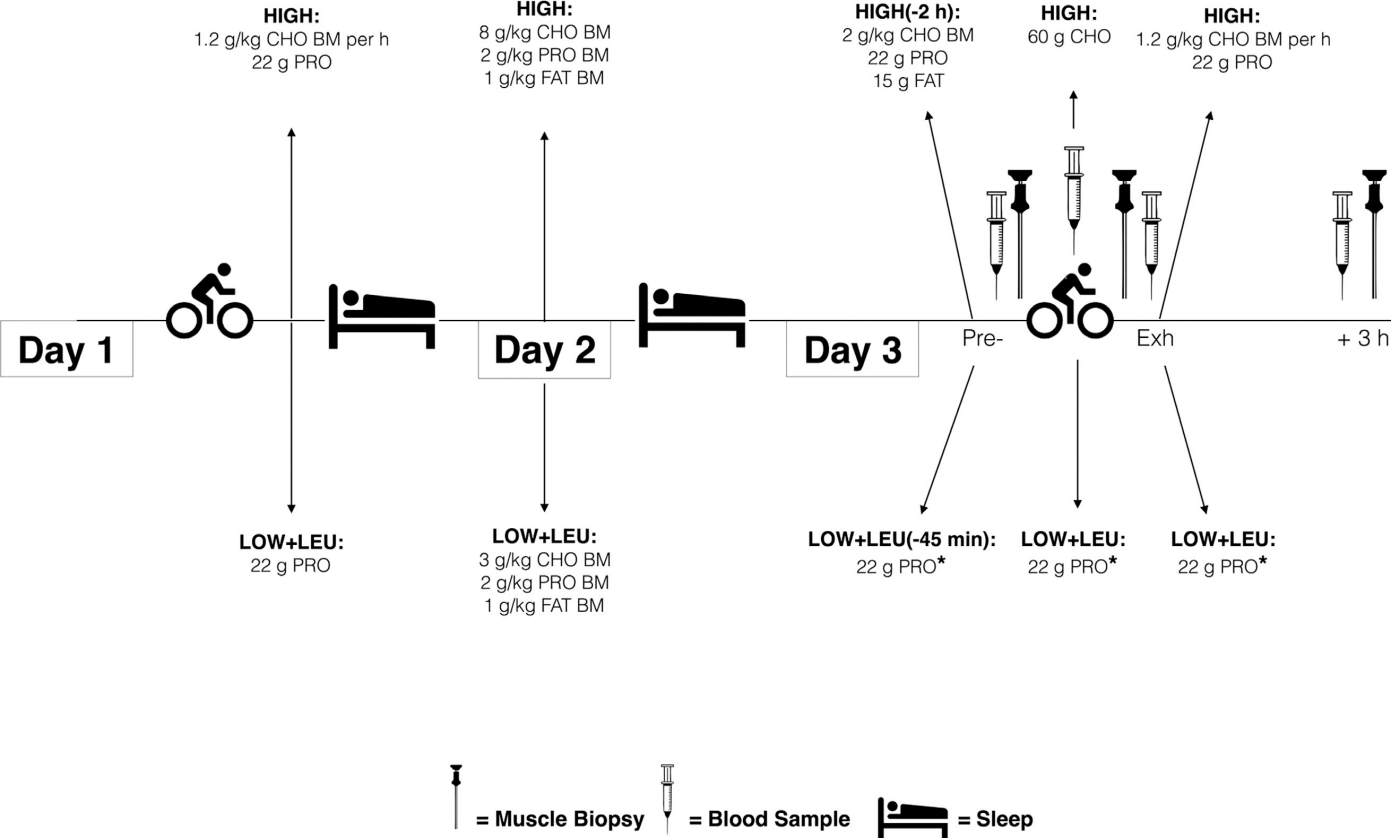
Schematic overview of the experimental protocol. On the evening of day 1, subjects completed a glycogen depleting protocol followed by 3 h of best practice recovery nutrition (HIGH) or sleep‐low (LOW + LEU). Throughout the entirety of day 2, subjects consumed a high CHO diet (HIGH) or alternatively, a low CHO and low energy dietary protocol (LOW + LEU) that was matched for both protein and fat intake. During the main experimental trial on day 3, subjects performed an exhaustive cycling protocol in conditions of best practice nutrition (HIGH) in which high CHO intakes were consumed before, during, and after exercise. In contrast, in the LOW + LEU trial, subjects consumed leucine‐enriched protein only. In this way, the LOW + LEU trial represented 3 days of an amalgamation of train‐low strategies consisting of sleep low (day 1), low dietary CHO intake (day 2), and omission of CHO intake before, during, and after exercise (day 3). Muscle biopsies were obtained immediately pre‐exercise, at the point of exhaustion (Exh) and at 3 h post‐exhaustion. *denotes leucine‐enriched protein.

In a repeated‐measures crossover design separated by 7–9 days, subjects completed two exhaustive exercise trials in conditions of high CHO availability (HIGH) or reduced CHO availability but with leucine‐enriched protein feeding before, during, and after exercise (LOW + LEU). At 36–40 h prior to the main experimental trials, all subjects performed a glycogen depletion protocol followed by 36 h of high or low CHO intake so as to manipulate pre‐exercise muscle glycogen content prior to the exhaustive exercise protocol. Subjects in HIGH then completed the exhaustive exercise protocol in conditions considered as best nutritional practice, that is pre‐exercise meal consisting of both CHO and protein, CHO intake during exercise and both CHO and protein intake post‐exercise. In contrast, subjects in LOW + LEU commenced the exhaustive exercise protocol with reduced pre‐exercise muscle glycogen and only consumed leucine enriched whey protein before, during, and after exercise. As such, this trial represented conditions of reduced CHO and absolute energy availability, but high protein availability throughout. Muscle biopsies were obtained from the vastus lateralis immediately before, post‐, and at 3 h post‐exercise. An overview of the experimental protocol is shown in Figure 1.

## Experimental Protocol

### Assessment of maximal oxygen uptake

At 7–10 days prior to the experimental trials, all participants were initially assessed for peak oxygen consumption (VO_2peak_) and peak aerobic power (PPO) as determined during an incremental cycle test performed on an electromagnetically braked cycle ergometer as described previously (Impey et al. 2015).

#### Day 1 – Glycogen depletion protocol

Participants arrived at the laboratory on the evening (1900 h) of the first experimental day having avoided alcohol and vigorous physical activity for the previous 24 h. Nude body mass was recorded and a heart rate (HR) monitor (Polar FT1, Finland) was fitted. Subjects then performed an intermittent glycogen‐depleting cycling protocol lasting ~120 min, as described previously by our group (Taylor et al. 2013). The activity pattern and total time to exhaustion were recorded, and water was consumed ad libitum throughout exercise. These parameters were repeated exactly during the second experimental condition and all cycling protocols were conducted on a fully adjustable electromagnetically braked cycle ergometer (SRM, Julich, Germany). Following the depletion exercise protocol, participants in HIGH consumed high carbohydrate at a rate of 1.2 g kg^−1^ body mass (BM) carbohydrate per hour for the first three hours in a mixture of bars and fluids (GO Bars/Go Energy, Science in Sport, Nelson, UK) and 22 g of whey protein immediately post‐exercise (REGO Protein, Science in Sport, Nelson, UK). In contrast, when participants completed the LOW + LEU trial, they consumed no carbohydrate within the 3 h recovery period, but did consume the same bolus of 22 g whey protein (REGO Protein, Science in Sport, Nelson, UK) immediately post‐exercise.

#### Day 2 – Nutritional protocols

During day 2, subjects in the HIGH trial consumed 8 g kg^−1^ BM carbohydrate as a mixture of foods and sports supplements designed to maximize muscle glycogen replenishment, whereas in the LOW + LEU trial, subjects consumed 3 g kg^−1^ BM carbohydrate in order to minimize muscle glycogen replenishment. In both trials, subjects also consumed 2 g kg^−1^ BM protein and 1 g kg^−1^ BM fat. In this way, it was expected that subjects would commence the main experimental trial on the morning of day 3 with high (HIGH) or low (LOW + LEU) muscle glycogen while also having consumed identical protein and fat intake and completed the same exercise loading patterns.

#### Day 3 – Main experimental trial

On the morning of the Day 3, subjects reported to the laboratory in a fasted state and an indwelling cannula (Safety Lock 22G, BD Biosciences, West Sussex UK) was inserted into the anticubital vein in the anterior crease of the forearm and a resting blood sample drawn. After a resting blood sample was taken, the cannula was flushed with ~5 mL of sterile saline (Kays Medical supplies, Liverpool, UK) to keep the cannula patent and sterile, this procedure was repeated after each subsequent blood draw. Following blood sampling, subjects in HIGH received a standardized breakfast containing 2 g kg^−1^ BM CHO and an absolute dose of 22 g whey protein and 15 g fat at 2 h prior to commencing the exhaustive exercise protocol. Two hours were provided postprandial so as to allow sufficient time for digestion prior to commencing exhaustive exercise. In contrast, subjects in the LOW + LEU trial consumed a leucine‐enriched protein drink that is not commercially available (Science in Sport, Nelson, UK) containing a total of 22 g protein of which 6.3 g was leucine at 45 min prior to commencing exercise. The leucine‐enriched protein was given 45 min prior to exercise (as opposed to 2 h prior) so as to ensure sufficient circulatory amino acid availability during the exercise protocol (Impey et al. 2015). Additionally, these timings and dosing strategies are in accordance with nutritional practices of professional road cyclists and as such, it was our aim to examine a real‐world nutritional protocol (J. P. Morton, unpubl. data). Subjects then completed a prescribed cycling protocol consisting of 4 × 30 sec high‐intensity intervals (HIT) at 150% PPO interspersed with 2.5 min active recovery at 40% PPO followed by 45 min steady state (SS) cycling at 50% PPO so as to examine the effects of altered substrate availability on standardized exercise responses (i.e., RPE, HR, substrate utilization). During the HIT and steady‐state component, subjects in HIGH ingested a 6% CHO solution providing 20 g of CHO (GO Electrolyte, Science in Sport, Nelson, UK) at 20 min intervals so as to provide 60 g in the first 60 min of exercise, thereby in accordance with current nutritional recommendations for CHO intake during exercise. Subjects in LOW + LEU ingested one‐third of the leucine‐enriched protein mix providing 7.3 g of protein at the same 20 min intervals as the feeding strategy in HIGH, so as to provide 22 g protein per hour in an attempt to maintain high circulatory amino acid availability during exercise (Impey et al. 2015). Following the steady‐state cycle, 5 min of active recovery was provided and subjects then commenced an exercise capacity test consisting of intermittent “1 min efforts” corresponding to 80% PPO interspersed with 1 min recovery periods at 40% PPO. This intermittent protocol was followed until the subjects reached volitional fatigue. Physiological and perceptual measures were recorded at regular intervals throughout exercise and substrate utilization was assessed during the steady‐state component of the exercise protocol using online gas analysis (CPX Ultima, Medgraphics, Minnesota) and the equations of Jeukendrup and Wallis (2005). At the point of exhaustion, subjects in HIGH received the same 1.2 g kg^−1^ BM CHO feeding strategy as following the depletion ride and an absolute dose of 22 g whey protein immediately after whereas subjects in LOW + LEU consumed 22 g of the leucine enriched protein only immediately after exercise (Fig. 1). In this way, subjects in HIGH undertook and recovered from exercise having ingested 6.3 g kg^−1^ BM CHO across the duration of the experimental trial along with a total of 44 g of protein, which contained 4.6 g of leucine. In comparison, subjects in LOW + LEU consumed a total of 66 g of protein of which 18.9 g was leucine across the duration of the main experimental trial. Laboratory conditions remained constant across all experimental trials (19–21°C, 40–50% humidity) and an overview of the nutritional feeding protocol is shown in Figure 1.

### Blood analysis

Blood samples were collected in vacutainers containing K_2_ EDTA, lithium heparin, or serum separation tubes, and stored on ice or at room temperature until centrifugation at 1500 g for 15 min at 4°C. Serum and plasma were aliquoted and stored at −80°C until analysis. Plasma glucose, lactate, FFA, glycerol, *β*‐hydroxybutyrate, and amino acids were analyzed as previously described (Impey et al. 2015).

### Muscle biopsies

Skeletal muscle biopsies were obtained from the vastus lateralis immediately before exercise, at the point of fatigue, and at 3 h post‐completion of the exercise protocol. Muscle biopsies were obtained from separate incision sites (2–3 cm apart) from the lateral portion of the vastus lateralis muscle. Biopsies were obtained using a Bard Monopty Disposable Core Biopsy Instrument (12 gage × 10 cm length, Bard Biopsy Systems, Tempe, AZ). Samples were obtained (approximately 50 mg) under local anesthesia (0.5% marcaine) and immediately frozen in liquid nitrogen and stored at −80°C for later analysis.

### RNA extraction and analysis

Muscle samples (~20 mg) were immersed and homogenized in 1 mL TRIzo (Thermo Fisher Scientific, UK). RNA was extracted according to the manufacturer's instructions. RNA concentration and purity were assessed by UV spectroscopy at ODs of 260 and 280 nm using a Nanodrop 3000 (Fisher, Rosklide, Denmark). A quantity of 70 ng RNA was used for each PCR reaction.

### Primer design

Primer sequences were identified using Gene (NCBI, http://www.ncbi.nlm.nih.gov.gene) and designed using Primer‐BLAST (NCBI, http://www.ncbi.nlm.nih.gov/tools/primer-blast). Sequence homology searches ensured specificity; all primers had no potential unintended targets following a blast search. The primers were ideally designed to yield products spanning exon–exon boundaries to prevent any amplification of gDNA. Three or more GC bases in the last five bases at the 3′ end of the primer were avoided. Secondary structure interactions (hairpins, self‐dimer, and cross dimer) within the primer were avoided. All primers were between 16 and 25 bp, and amplified a product of between 141 and 244 bp; primers were purchased from Sigma (Suffolk, UK).

### Reverse transcriptase quantitative Real‐Time Polymerase Chain Reaction (RT‐qRT‐PCR)

RT‐qRT‐PCR amplifications were performed using QuantiFast^TM^ SYBR^®^ Green RT‐PCR one‐step kit on a Rotogene 3000Q (Qiagen, Crawley, UK) supported by rotogene software (Hercules, CA). RT‐qTR‐PCR was performed as follows: hold 50°C for 10 min (reverse transcription/cDNA synthesis), 95°C for 5 min (transcriptase inactivation and initial denaturation step), and PCR steps of 40 cycles; 95°C for 10 sec (denaturation), 60°C for 30 sec (annealing and extension). Upon completion, dissociation/melting curve analyses were performed to reveal and exclude nonspecific amplification or primer–dimer issues (all melt analysis in this study presented single reproducible peaks for each target gene suggesting amplification of a single product). Following initial screening of suitable housekeeping genes, GAPDH showed the most stable *C*_t_ values across all RT‐PCR runs, subjects, and regardless of experimental condition (25.5 ± 1.01), and was selected as the reference gene in all RT‐PCR assays. The relative gene expression levels were calculated using the comparative *C*_t_ (^ΔΔ^ *C*_t_) equation (Schmittgen and Livak 2008) where the relative expression was calculated as 2^−ΔΔct^ a where *C*_t_ represents the threshold cycle. mRNA expression for all target genes was calculated relative to the reference gene (GAPDH) within same subject and condition and to a calibrator of HIGH condition pre‐exercise. Gene expression responses were not quantified immediately post‐exercise given that many target genes are only up‐regulated in the hours in recovery from exercise.

### Muscle glycogen concentration

Muscle glycogen concentration was determined according to the acid hydrolysis method described by van Loon et al. (2000) with glucose concentration quantified using a commercially available kit (GLUC‐HK, Randox Laboratories, Antrim, UK). Glycogen concentration is expressed as mmol kg^−1^ dry weight (dw) and intra‐assay coefficients of variation was <5%.

### [*γ* ^−32^P] ATP kinase assay

A quantity of 30 mg of muscle tissue was used for the measurement of p70S6K1 and AMPK*α*1 and *α*2 activity as previously described (McGlory et al. 2014).

### Statistics

All statistical analyses were performed using Statistical Package for the Social Scientist (SPSS version 21). Descriptive statistics were produced for all data sets to check for normal distribution indicated by Kolmogorov–Smirnov. Changes in exercise capacity were analyzed using Student t‐test. Changes in physiological and molecular responses between conditions (i.e., muscle glycogen, circulatory metabolites, amino acids, mRNA transcription, and activity of signaling molecules) were analyzed using two‐way repeated‐measures general linear model, where the within factors were time and condition. If Mauchley's test of sphericity indicated a minimum level of violation, as assessed by a Greenhouse Geisser epsilon (*ε*) of ≥0.75, data were corrected using the Huynh‐Feldt *ε*. If Mauchley's test of sphericity was violated, data were corrected using Greenhouse Geisser *ε*. Where a significant main effect was observed, pairwise comparisons were analyzed according to Bonferroni post hoc tests in order to locate specific differences. An alpha value of *P *<* *0.05 was utilized for all tests and all data in text, figures, and tables are presented as mean ± SD.

## Results

### Skeletal muscle glycogen content and exercise capacity

**Figure 2:**
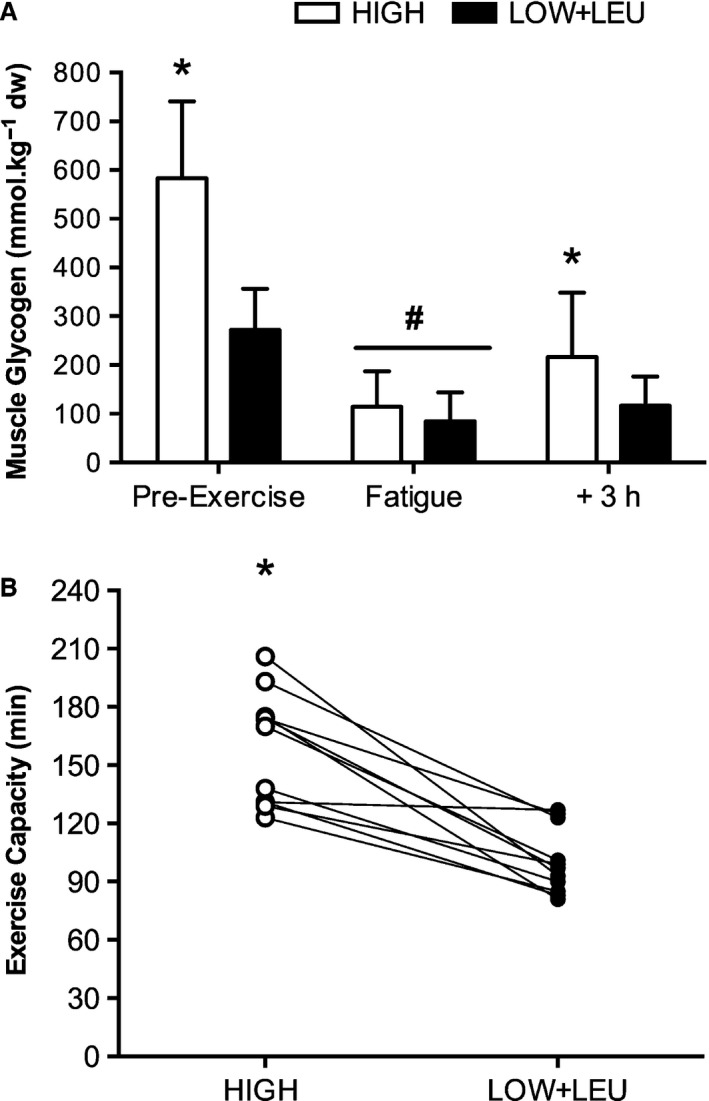
(A) Skeletal muscle glycogen content, (B) Exercise capacity (reflective of set work protocol plus time to exhaustion). *P < 0.05, significant main effect of condition. ^**#**^ *P *<* *0.05, significant effect of exercise.

Muscle glycogen content was significantly higher pre‐exercise (*P* = 0.003) in the HIGH trial compared with the LOW + LEU trial (Fig. 2A). Exhaustive exercise also significantly reduced (*P* < 0.001) muscle glycogen stores to comparable levels (<120 mmol kg^−1^ dw) with no difference observed between conditions (*P* = 0.278). As expected, post‐exercise CHO feeding significantly increased (*P* = 0.003) muscle glycogen in the HIGH trial to approximately 200 mmol kg^−1^ dw, whereas no glycogen resynthesis was observed in the LOW + LEU trial. In accordance with distinct differences in CHO availability, exercise capacity was significantly greater (*P* < 0.001) in the HIGH trial (158 ± 28 min) compared with the LOW+LEU trial (100 ± 17 min), an effect that was apparent in all 11 subjects (range: 4–113 min, 95% CI differences: 38–79 min) (Fig. 2B).

### Physiological and metabolic responses to exercise

**Figure 3:**
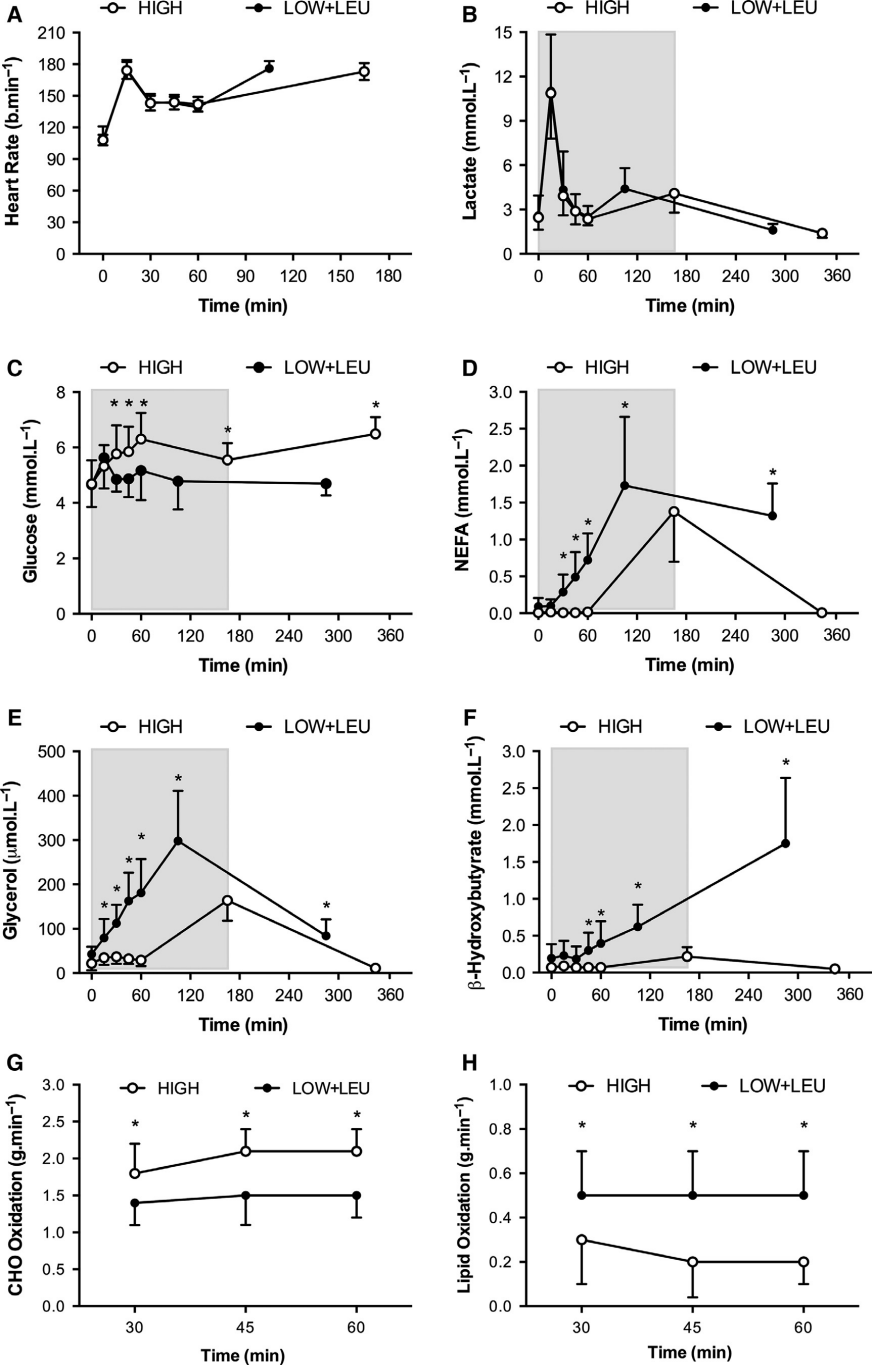
(A) Heart rate response during exercise and plasma (B) Lactate, (C) Glucose, (D) NEFA, (E) Glycerol, (F) *β*‐hydroxybutyrate, before, during, and after exercise. (G) Carbohydrate oxidation and (H) Lipid oxidation during exercise. *P <* *0.05, significant main effect of condition. Shaded area represents exercise duration.

Subject's heart rate (*P* = 0.458) and plasma lactate (*P* = 0.929) during exercise did not display any significant differences between trials (Fig. 3A and B, respectively). However, in accordance with differences in CHO availability, plasma glucose was significantly lower (*P* = 0.007) in the LOW + LEU trial when compared with the HIGH trial (Fig. 3C). In contrast, plasma NEFA (*P* < 0.001), glycerol (*P* < 0.001), and *β*‐OHB (*P* < 0.001) were all significantly elevated during exercise in the LOW + LEU trial compared with the HIGH trial (Fig. 3D, E and F, respectively). As a result of such differences in substrate availability, the pattern of fuel use during the steady‐state component of the exercise protocol was different such that subjects in LOW+LEU oxidized less CHO (*P* = 0.004) and more lipid (*P* = 0.007) when compared with the HIGH trial (Fig. 3G and H, respectively).

### Regulation of mitochondrial biogenesis‐related cell signaling

**Figure 4:**
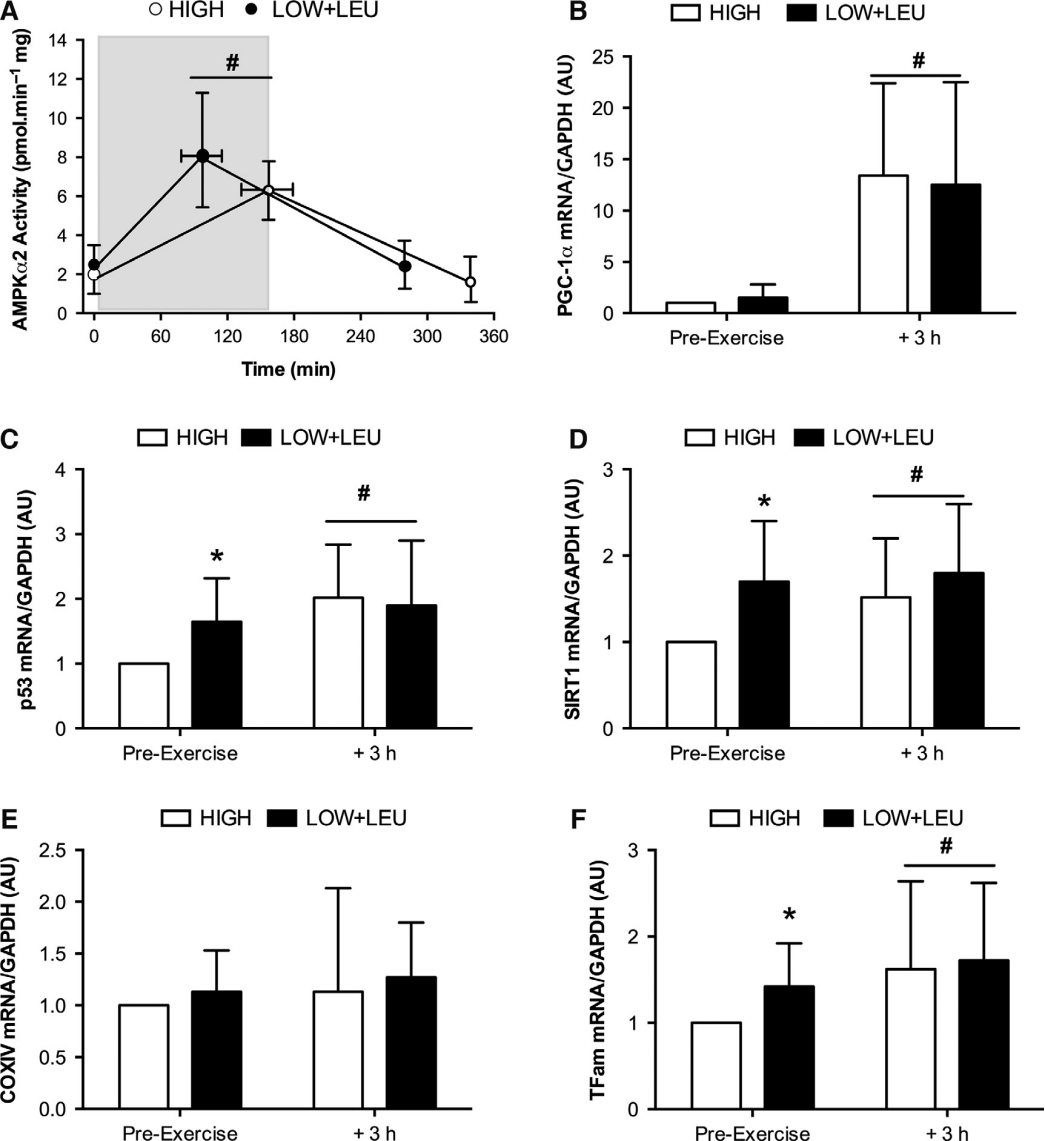
(A) AMPK *α*2 activity pre‐, post‐, and 3 h post‐exercise. Shaded area represents exercise duration. (B) PCG‐1*α*, (C) p53, (D), SIRT1, (E) COXIV, and (F) Tfam mRNA pre‐ and 3 h post‐exercise. *P <* *0.05, significant main effect of condition, ^**#**^ *P < *0.05, significant main effect of exercise.

The exhaustive exercise protocol did not increase AMPK*α*1 activity in either the HIGH or LOW trial (data not shown). In contrast, exhaustive exercise significantly increased AMPK*α*2 activity by approximately 4‐fold immediately post‐exercise (*P* = 0.001) though no difference was observed between conditions (Fig. 4A). Similarly, PGC‐1*α* mRNA (*P* = 0.007) was significantly increased to comparable levels between trials at 3 h post‐exercise with no differences between conditions (Fig. 4B). p53 (*P* = 0.01), SIRT1 (*P* = 0.007), and Tfam (*P* = 0.029) mRNA were significantly elevated pre‐exercise in the LOW + LEU condition compared with the HIGH trial (Fig. 4C, D and F, respectively). Acute exercise also significantly increased p53 (*P* = 0.013), Tfam (*P* = 0.038), and SIRT1 (*P* = 0.013) mRNA to comparable levels at 3 h post‐exercise such that no differences were apparent between conditions. In contrast, neither nutrient availability nor the exhaustive exercise protocol affected COXIV mRNA levels (Fig. 4F).

### Plasma amino acid levels

**Figure 5:**
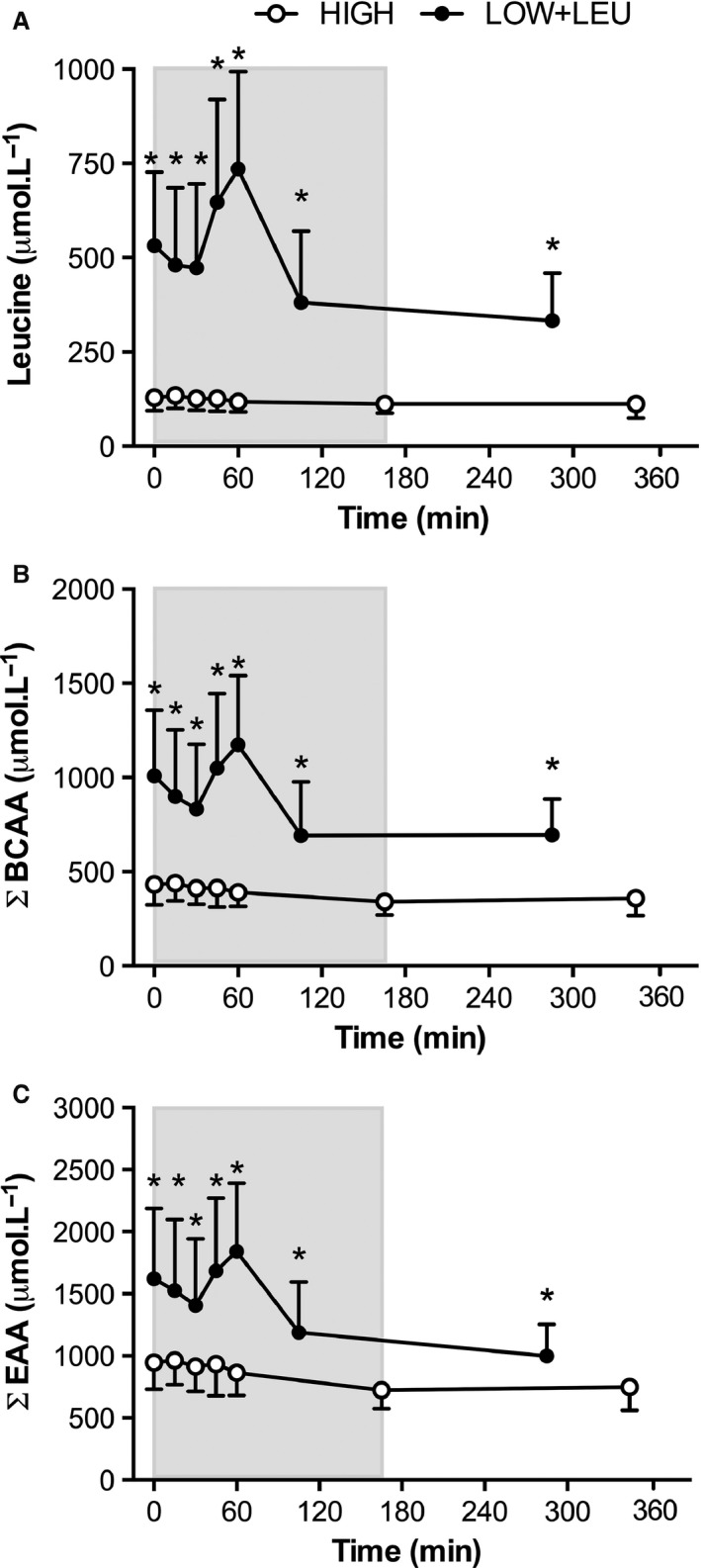
(A) Leucine, (B) BCAAs, and (C) EAAs before, during, and after exercise. Shaded area represents exercise duration. *P <* *0.05, significant main effect of condition.

Plasma leucine (*P* < 0.001), BCAA (*P* < 0.001), and EAA (*P* < 0.001) concentrations were all significantly elevated (Fig. 5A, B, and C, respectively) before, during, and after exercise in the LOW + LEU trial compared with the HIGH trial.

### Regulation of protein synthesis and breakdown‐related cell signaling

**Figure 6:**
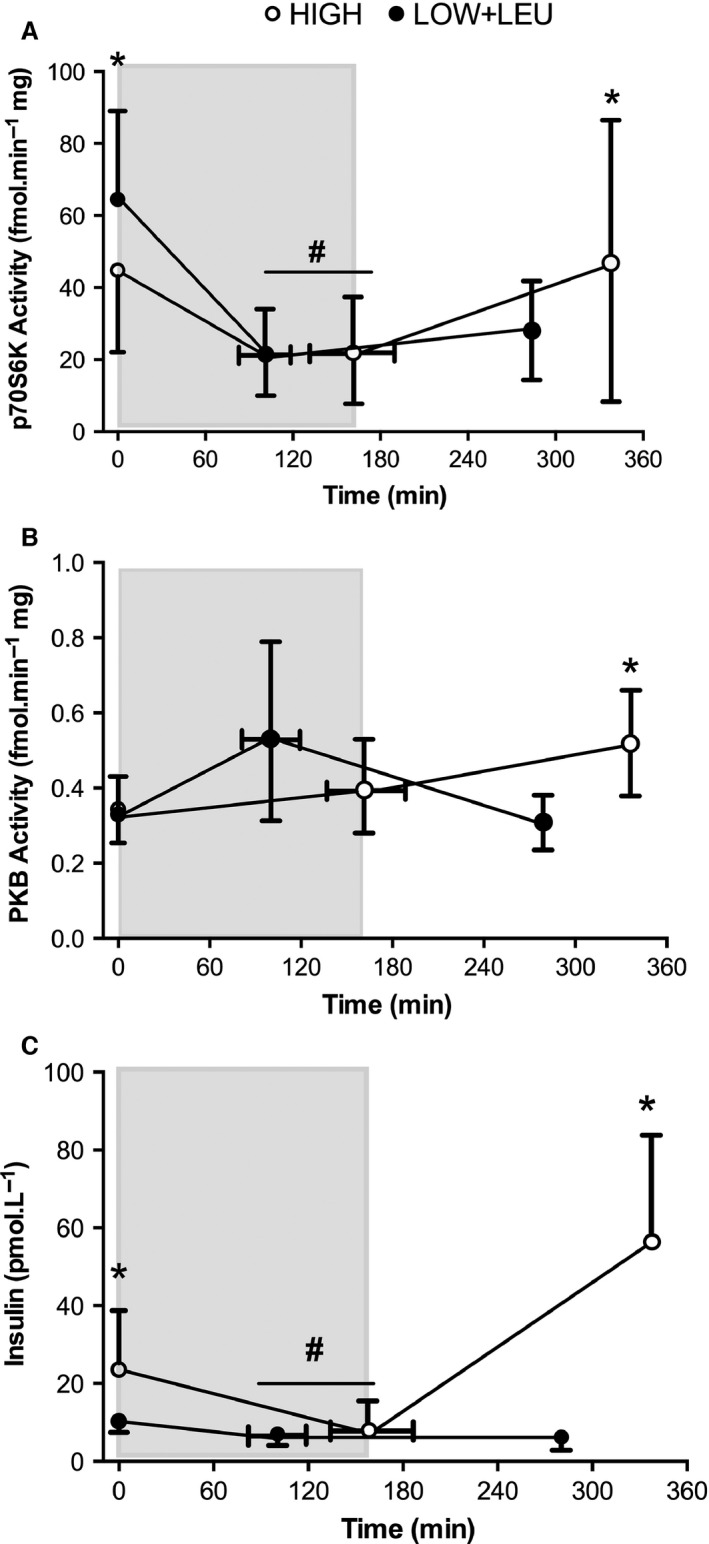
(A) p70S6K, (B) PKB activity, and (C) Serum insulin pre‐, post‐, and 3 h post‐exercise. Shaded area represents exercise duration. *P <* *0.05, significant main effect of condition, ^**#**^ *P *<* *0.05, significant main effect of exercise.

**Figure 7:**
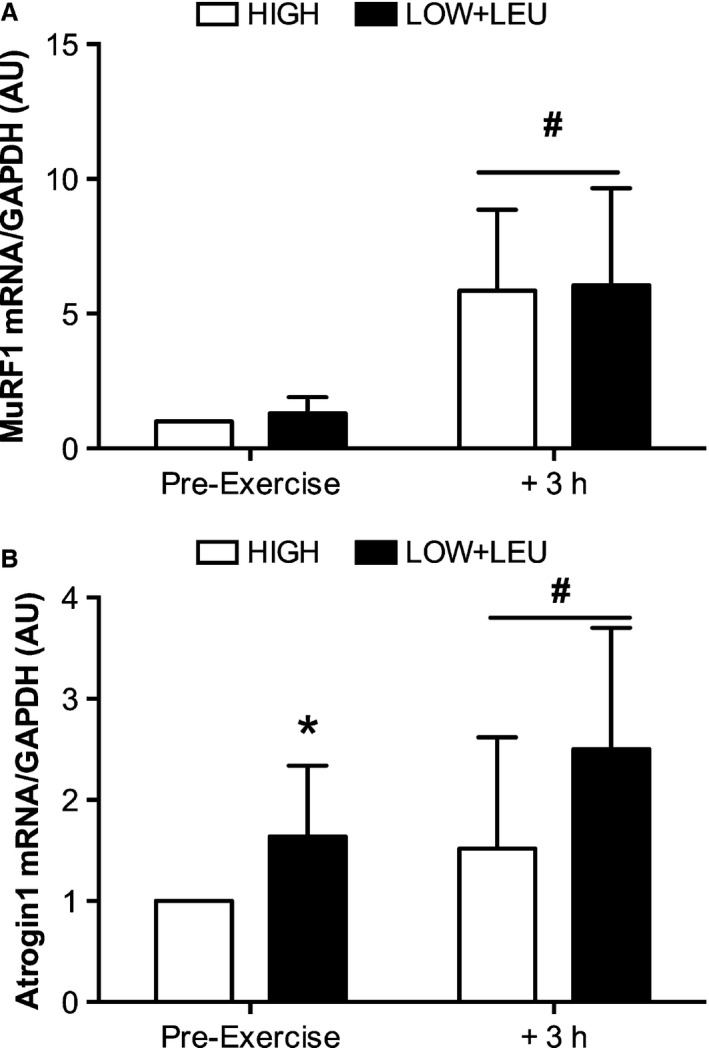
(A) MuRF1 and (B) Atrogin1 mRNA pre‐ and 3 h post‐exercise. *P <* *0.05, significant main effect of condition, ^**#**^ *P *<* *0.05, significant main effect of exercise.

p70S6K activity was significantly elevated (*P* = 0.03) immediately prior to exercise in the LOW + LEU trial compared with the HIGH condition (Fig. 6A). Following exhaustive exercise, p70S6K activity was suppressed (*P* < 0.001) to comparable levels in both the LOW + LEU and HIGH conditions. In contrast to pre‐exercise status, p70S6K activity at 3 h post‐exercise was significantly higher (*P* = 0.04) in the HIGH trial compared with the LOW + LEU trial. PKB activity tended to increase (*P* = 0.056) immediately post‐exercise in both groups (Fig. 6B). However, at 3 h after exercise, PKB activity was significantly greater in the HIGH trial compared with the LOW + LEU condition (*P* = 0.021). Consistent with the feeding of CHO before and after exercise, plasma insulin levels were significantly higher immediately pre‐ and 3 h post‐exercise (*P* < 0.05) in the HIGH trial compared with the LOW + LEU trial (Fig. 6C), whereas exhaustive exercise reduced insulin levels (*P* = 0.001) to comparable levels between trials. Atrogin1 mRNA expression was higher immediately (*P* = 0.009) before exercise in the LOW + LEU trial compared with the HIGH trial, whereas pre‐exercise nutrient status had no effect on MuRF1 mRNA content (Fig. 7A and B, respectively). Exhaustive exercise also increased (*P* < 0.05) the expression of both genes at 3 h post‐exercise to comparable levels in the LOW + LEU and HIGH trials.

## Discussion

The aim of this study was to assess the effects of high CHO versus low CHO availability on the modulation of those skeletal muscle cell‐signaling pathways with putative roles in the regulation of both mitochondrial biogenesis and muscle protein synthesis. We adopted a train‐low model that represents an amalgamation of previously studied train‐low paradigms (incorporating both CHO and energy restriction) and that we consider representative of the real‐world practices often adopted by elite level endurance athletes. Confirming our hypothesis, we provide novel data by demonstrating that although reduced CHO availability impaired exercise capacity, our CHO restriction protocol induced comparable AMPK‐PGC‐1*α* signaling, thus inducing “work‐efficient” signaling responses. In contrast, refraining from CHO intake in the post‐exercise period (despite the intake of leucine‐rich protein) maintains p70S6K activity at basal levels. When taken together, these data have practical implications by suggesting that: (1) CHO restriction before and during exercise induces work‐efficient cell signaling related to mitochondrial biogenesis but furthermore; (2) complete CHO and energy restriction in the post‐exercise period reduces the activation of key signaling proteins regulating skeletal muscle modeling processes. As such, these data suggest that although athletes may benefit from carefully scheduled periods of reduced CHO availability before and during exercise so as to enhance mitochondrial‐related cell signaling, they should consume *both* CHO and protein post‐exercise so as to restore the activation of MPS‐related signaling. Furthermore, our data lend support for a potential “fuel for the work required” train‐low paradigm in which athletes could strategically reduce CHO availability prior to completing predetermined training workloads that can be readily performed with reduced CHO availability, thereby inducing a “work‐efficient” approach to training. Alternatively, when the goals of the training session are to complete the highest workload possible over more prolonged durations, then adequate CHO should be provided in the 24 h period prior to and during the specific training session.

To achieve our model of CHO restriction, we employed a protocol consisting of an amalgamation of previously studied train‐low models. For example, having completed an initial glycogen depletion protocol on the evening of day 1, subjects adhered to a dietary protocol consisting of reduced CHO and energy intake for the subsequent 36 h prior to arriving at the laboratory on the morning of day 3 for the main experimental trial. This initial approach is thus representative of an acute sleep‐low model (i.e., CHO restriction in the initial hours following completion of the depletion protocol on day 1) followed by consumption of a low CHO diet for the entirety of day 2. This approach was successful in inducing marked differences in pre‐exercise muscle glycogen content (≈600 mmol kg^−1^ dw vs. 300 mmol kg^−1^ dw in HIGH and LOW + LEU, respectively) on the morning of the main experimental trial commenced on day 3 (see Fig. 2). Consistent with the effects of post‐exercise CHO restriction on regulation of gene expression (Pilegaard et al. 2005; Bartlett et al. 2013; Lane et al. 2015), we also observed marked effects on the expression of genes associated with regulatory roles in mitochondrial biogenesis. Indeed, subjects arrived at the laboratory on the morning of day 3 with higher pre‐exercise mRNA content of p53, SIRT1, and Tfam in the LOW + LEU trial (see Fig. 4), all of which are thought to be regulators of mitochondrial biogenesis (Gurd et al. 2010; Saleem et al. 2013; Bartlett et al. 2014, 2015). The observation of enhanced p53 mRNA following post‐exercise CHO restriction is consistent with previous data from our group demonstrating that the exercise‐induced phosphorylation of p53^Ser15^ is enhanced with CHO restriction before, during, and after exercise (Bartlett et al. 2013). On this basis, it is possible that sustained p53 activation in recovery from the glycogen depletion exercise protocol in conjunction with no CHO intake in the LOW + LEU trial may be a common signaling axis regulating the aforementioned gene expression responses. Indeed, p53 is known to autoregulate its own expression (Deffie et al. 1993) as well as both basal‐ and exercise‐induced Tfam mRNA levels (Saleem et al. 2013). Furthermore, previous work in cell culture models of nutrient deprivation has shown transcriptional regulation of SIRT1 is mediated through p53 binding to the SIRT1 promoter (Nemoto et al. 2004).

In relation to the main experimental trial on day 3, subjects also refrained from CHO intake before, during, and after the exercise protocol (but consumed leucine‐rich protein), thereby representative of an amalgamation of “fasted (i.e. CHO restricted)” train‐low protocols (Van Proeyen et al. 2011) and “protein only” training sessions (Taylor et al. 2013; Impey et al. 2015). As expected, we observed distinct differences in substrate availability and fuel utilization during the steady‐state exercise protocol, as evidenced by *β*‐OHB levels comparable with nutritional ketosis (Cox and Clark 2014), as well as greater levels of circulating NEFA, glycerol, and lipid oxidation in the LOW+LEU trial compared with the HIGH trial. Consistent with the well‐documented effects of both endogenous and exogenous CHO availability on exercise performance (Hawley et al. 1997; Stellingwerff and Cox 2014), we also observed reduced exercise capacity in our LOW+LEU trial compared with the HIGH trial, an effect that was evident in all eleven subjects. Such data therefore reiterate the obvious necessity for high CHO availability before and during those training sessions in which prolonged high‐intensity workloads are required to be completed.

From a biochemical perspective, it is well‐accepted that reduced muscle glycogen stores induce greater skeletal muscle cell signaling when compared with “matched‐work” exercise protocols (i.e., completion of identical duration and intensity) undertaken with high glycogen stores (Bartlett et al. 2015). For example, AMPK^Thr172^ phosphorylation (Yeo et al. 2010; Lane et al. 2015), ACC^Ser79^ phosphorylation (Bartlett et al. 2013), AMPK‐*α*2 activity (Wojtaszewski et al. 2003), and the nuclear abundance of AMPK‐*α*2 protein content (Steinberg et al. 2006) are all up‐regulated to a greater extent when acute exercise is completed with reduced pre‐exercise muscle glycogen stores, an effect likely mediated via the presence of a glycogen‐binding domain on the *β*‐subunit of the AMPK heterotrimer (McBride et al. 2009). Furthermore, exogenous CHO feeding during exercise attenuates AMPK activity only when muscle glycogen sparing has occurred (Akerstrom et al. 2006). The present data extend these findings as we show for the first time comparable cell signaling effects despite the completion of significantly less work completed. Indeed, although we observed no effects of exercise on AMPK‐*α*1 activity (as reported by others, Fuji et al. 2000), we observed similar increases in AMPK‐*α*2 activity and PGC‐1*α* mRNA expression at comparable levels of absolute glycogen, despite mean differences of 60 min less work completed in LOW + LEU versus HIGH conditions. Such data therefore support the “glycogen threshold” hypotheses (Philp et al. 2012) surmising that a critical absolute level of glycogen must be exceeded in order for significant AMPK signaling to occur during prolonged endurance exercise protocols.

Given the effects of reduced CHO and energy deficit on muscle protein degradation and protein synthesis (Lemon and Mullin 1980; Howarth et al. 2010; Pasiakos et al. 2010, 2013; Areta et al. 2014), we also chose to feed leucine‐enriched whey protein before, during, and after the main experimental trial on day 3. In accordance with the role of leucine availability in modulating MPS (Karlsson et al. 2006; Churchward‐Venne et al. 2014), we observed higher pre‐exercise p70S6K activity in our LOW + LEU trial when compared with the HIGH trial, though we acknowledge that differences in timing of pre‐exercise feeding between trials may also have contributed to this finding. The effects of acute endurance exercise on regulation of p70S6K are not well established (and are typically limited to measures of phosphorylation status) with some studies reporting increases (Mascher et al. 2007, 2011) and others, no change (Coffey et al. 2006; Vissing et al. 2013). Nevertheless, consistent with the notion that exercise suppresses MPS during exercise (Rennie et al. 1980; Rose and Richter 2009), we observed significant reductions in p70S6K activity immediately post‐exercise to almost identical levels between trials. To the best of our knowledge, we are only the second group to directly quantify p70S6K activity in response to endurance type exercise protocols and our data conflict with Apro et al. (2015) who observed no change in response to 5 × 4 min cycling at 85% VO_2max_. Such discrepancies between studies are likely most related to the exhaustive and energy restricting nature of our exercise protocol. While it is difficult to directly compare the total energy expenditure between this study and the data of Apro et al. (2015), the exercise interventions used here elicited considerably lower muscle glycogen concentrations of ~100 mmol kg^−1^ dw versus 350 mmol kg^−1^ dw in the study of Apro et al. (2015). The mechanism(s) behind suppressed p70S6K activity following exhaustive exercise likely involve a large number of signaling mechanisms and regulators within skeletal muscle. Indeed, AMPK mediated inhibition of mTOR through TSC2 (Sanchez et al. 2012) or via interaction with v‐ATPase‐Ragulator at the late endosomal/lysosme surface (Zhang et al. 2014), as well as p53‐REDD1‐mediated inhibition of mTOR (Feng and Levine 2010; Keller et al. 2013) are all potential candidates. Further studies are now required to test these hypotheses in human skeletal muscle.

In relation to post‐exercise feeding, we observed that the co‐ingestion of carbohydrate and whey protein feeding was sufficient to rescue p70S6K activity in the HIGH trial, whereas p70S6K remained suppressed in the LOW + LEU trial despite the intake of “leucine‐enriched” whey protein feeding. While it is difficult to readily ascertain the precise mechanism(s) underpinning these data, it is noteworthy that we did observe increased upstream signaling of PKB (Akt) in our HIGH condition, an effect that may be simply related to insulin‐mediated activation given the repeat 90 g intakes of CHO immediately post and at 1 and 2 h post‐exercise. In addition, the potential reduced PKB‐mediated activation of p70S6K may be due to the presence of low muscle glycogen per se given previous data demonstrating that the post‐exercise (albeit in resistance exercise) activation of Akt phosphorylation is suppressed (independent of post‐exercise feeding) when muscle glycogen levels are comparable to that observed in this study, that is <150 mmol kg^−1^ dw (Creer et al. 2005). Alternatively, the reduced activation of p70S6K in the LOW + LEU trial could be due to high circulating NEFA concentrations given that high fat availability can impair MPS. Indeed, infusion of intralipid and heparin to elevate circulating NEFA concentrations (to comparable levels seen here) attenuates MPS in human skeletal muscle in response to ingesting 21 g amino acids under euglycemic hyperinsulemic clamp conditions (Stephens et al. 2015). Furthermore, Kimball et al. (2015) also reported that high fat feeding impairs MPS in rat liver in a manner associated with reduced p70S6K phosphorylation (but not PKB phosphorylation).

The functional relevance of such divergent responses cannot be ascertained from this study given that we did not directly quantify MPS. Indeed, previous data have suggested that low muscle glycogen availability (during resistance exercise protocols) does not have any measurable effect on post‐exercise MPS (Camera et al. 2012), whereas the recovery from endurance exercise when glycogen levels remain low results in negative protein balance (Howarth et al. 2010). As noted previously, however, the experimental protocol adopted here was a deliberate manipulation of both CHO and energy availability. Indeed, consistent with the effects of acute energy deficit on skeletal muscle proteolysis (Carbone et al. 2013), we also observed increased resting mRNA expression of Atrogin 1 in the LOW + LEU trial when compared with the HIGH trial. Furthermore, given that acute energy deficit also impairs MPS in a manner associated with reduced PKB (Pasiakos et al. 2010) and p70S6K phosphorylation (Pasiakos et al. 2013), the divergent signaling responses observed here may indeed manifest as functional reductions in MPS and negative protein balance. From a practical perspective, our data suggest that while there may be benefits of restricting CHO intake in the post‐exercise period in terms of enhancing mitochondrial signaling, it is also necessary to consume sufficient CHO intake in the immediate post‐exercise period so as to replenish muscle glycogen to sufficient levels per se and/or obtain upstream signaling effects associated with feeding, the result of which could maintain the activity of those signaling proteins with putative roles in regulating MPS and skeletal muscle remodeling.

It is, of course, beyond the scope of the present paper to offer definitive guidelines on how best to periodize CHO restriction into an overall athletic training program. Nevertheless, in accordance with the work of Lane et al. (2015), we consider the signaling responses observed herein to offer further mechanistic support for the performance improvements observed by Marquet et al. (2016) while adopting the 3‐week sleep low‐training paradigm. In essence, the theme that emerges appears to be the concept of both “day‐to‐day” and “meal‐by‐meal” CHO periodization in accordance with the upcoming training workloads that have been prescribed. In practice, this approach of forward planning could represent an amalgamation of train‐low paradigms and is perhaps best communicated by the principle of “fuel for the work required”. Careful day‐to‐day periodization (as opposed to chronic periods of CHO restriction) is likely to maintain metabolic flexibility and still allow for the completion of high‐intensity and prolonged duration workloads on heavy training days, for example, interval type workouts undertaken above lactate threshold. Intuitively, train‐low sessions may be best left to those training sessions in which the intensity and duration of the session is not likely to be compromised by reduced CHO availability, for example, steady‐state type training sessions performed at intensities below the lactate threshold.

In summary, we have utilized an amalgamation of previously studied train‐low paradigms (considered representative of real‐world athletic practice) to demonstrate for the first time that CHO restriction before and during exhaustive exercise induces “work‐efficient” cell signaling related to mitochondrial biogenesis. However, in the absence of CHO feeding and absolute energy intake in the 3 h post‐exercise period, p70S6K activity remains suppressed despite consuming leucine‐rich protein immediately post‐exercise. When taken together, these data allude to a potential muscle glycogen threshold hypothesis surmising that reduced pre‐exercise muscle glycogen may not only enhance the activation of those pathways regulating mitochondrial biogenesis but also suggest that keeping glycogen and energy intake at critically low levels may impair the regulation of post‐exercise muscle protein synthesis. Furthermore, our data lend support for a potential “fuel for the work required” train‐low paradigm in that athletes could strategically reduce CHO availability prior to completing predetermined training workloads that can be readily performed with reduced CHO availability, thereby inducing a “work‐efficient” approach to training. Alternatively, when the goals of the training session are to complete the highest workload possible over more prolonged durations, then adequate CHO should be provided in the 24 h period prior to and during the specific training session. Future studies should now examine the functional relevance of the signaling responses observed here, not only in terms of acute muscle protein synthesis but also the chronic skeletal muscle and performance adaptations induced by long‐term use of this feeding strategy.

## Conflict of Interests

None declared.
